# A validation study for wide-range remote assessment of cognitive functions in the healthy older Japanese population: a pilot randomised crossover trial

**DOI:** 10.1186/s12877-023-04275-5

**Published:** 2023-09-19

**Authors:** Yuki Takakura, Mika Otsuki, Ryo Takagi, Kiyohiro Houkin

**Affiliations:** 1 Faculty of Health Sciences, Hokkaido University, N-12,W-5, Kita-Ku, Sapporo, 060-0812 Japan; 2 Institute of Health Science Innovation for Medical Care, Hokkaido University Hospital, Sapporo, Hokkaido Japan; 3 Emeritus Professor, Hokkaido University, Sapporo, Hokkaido Japan

**Keywords:** Tele-neuropsychology, Neuropsychological tests, Validation study, Cognitive functions, Older population

## Abstract

**Background:**

The assessment of a wide range of cognitive functions using video teleconference (VTC) systems cannot be applied in practice yet. We aimed to determine the feasibility and reliability of previously unvalidated remote cognitive function tests in Japan using common information and communication technology (ICT) devices, software, and VTC systems compared with face-to-face (FTF) assessment.

**Methods:**

The sample consisted of 26 participants from senior citizens clubs and an employment service centre in Sapporo Japan, including 11 females and 15 males (age averaged 78.6 ± 6.8 years). Tests included the RCPM, Story recall, 10/36 spatial recall, selective reminding test, SDMT, PASAT, FAB, TMT-A, TMT-B, visual cancellation task, digit span, tapping span. The experimental design was a counterbalanced crossover randomised controlled trial. Intraclass correlations (ICCs), paired-samples t-tests, Cohen’s Kappa (κ) coefficients, and Wilcoxon signed-rank test were calculated to compare the scores between VTC and FTF assessments.

**Results:**

All ICCs were significant and ranged from 0.47 (RCPM time) to 0.92 (RCPM score and PASAT), with a mean ICC of 0.75. Digit span using Cohen’s Kappa (κ) coefficient was significant, but the tapping span was not. Paired samples t-test showed statistically significant differences in SDMT, RCPM time, and cancellation time.

**Conclusions:**

The results suggest that remote video conference-based neuropsychological tests even using familiar devices and software may be able to assess a wide range of cognitive functions in the Japanese older population. As for the processing speed tasks, we need to create our own standards for the remote condition. For the tapping span, we should consider increasing the number of trials.

## Background

The number of people with age-associated cognitive impairment is increasing rapidly [1]. There are more than 55 million people with dementia worldwide, with nearly 10 million new cases reported every year [2]. Therefore, dementia is one of the most pressing health care issues worldwide [3].

The strategy for dementia in Japan is important worldwide because Japan has the highest proportion of older people in the world. Early detection and therapeutic intervention for aging people before the onset of dementia are important [4, 5]. For the early detection of dementia, both brain imaging and neuropsychological tests to assess cognitive function are essential.

However, there is an imbalance between the number of professionals who can administer detailed neuropsychological assessments and the number of older people who need such assessments [4, 5]. In addition, the concentration of the population in urban areas has caused problems in accessing medical care in remote rural areas due to a shortage of specialists [4].

Remote neuropsychological assessment using video teleconferencing (VTC) systems has been proposed as a potential solution [1, 4, 5]. In recent years, the COVID-19 pandemic has accelerated research on remote assessments [6, 7]. Inter-organisational guidelines for tele-neuropsychology have also been published in response to the COVID-19 pandemic [8]. Sufficient evidence has been accumulated to support the validity of VTC for remote neuropsychological assessment in older populations. In addition, VTC can be useful as a safe method for neuropsychological assessments of hospitalised patients during the COVID-19 pandemic and other situations requiring infection prevention [9].

Internationally, the validity of several types of remote tests using VTC has been reported [10–12]. However, there is a shortage of validation studies on the remote assessment of intellectual functions, processing speed, executive functions, and motor-dependent tasks using VTC [11, 12]. Therefore, the assessment of a wide range of cognitive functions using VTC cannot be applied in practice. Only four screening tests have been investigated in Japan for remote test reliability: the Revised Hasegawa’s Dementia Scale (HDS-R) [13], clock drawing task [14], Japanese version of the Montreal Cognitive Assessment (MoCA-J) [4], and the Alzheimer’s Disease Assessment Scale-cognitive component (ADAS-cog) [5].

Furthermore, because the equipment specialised for VTC used for validation in previous studies are expensive in Japan [4, 5, 13, 14], it is difficult to implement the same in clinical situations. Thus, there is a requirement for investigating the validity of VTC assessments using more common equipment and software to enable their clinical implementation. Therefore, the aims of this study were as follows: (1) to assemble a test battery that can assess a wide range of cognitive functions, such as intelligence, memory, attention, and frontal lobe functions, including motor-dependent tasks, and (2) to evaluate the reliability of these VTC tests using common equipment and software compared with face-to-face (FTF) assessment.

## Methods

### Participants

Twenty-six physically and mentally healthy Japanese adults without any history of neurological diseases participated in the study. They were recruited via senior citizen clubs and an employment service centre for independently living older people in Sapporo, Japan, from October 2019 to February 2020. The participants consisted of 11 women and 15 men aged between 65 and 84 years (mean age: 74.2, standard deviation [SD]: 5.2). Their average number of years of education was 12.8 (SD: 2.2, range: 9–16) (Table 1). We judged them to be in good physical and mental health based on interviews about their medical and life histories, and that they routinely work at the client sites through the employment service centre or participate in the citizen clubs. The total number of entrants was 27, but we excluded one participant because of a history of cerebrovascular disease. These participants were distinguished from those recruited to examine the reliability of the self-created test.

**Table 1:** Demographics of the validation study for remote assessment (*n* = 26)

Variable	Mean	SD	Range
Age (years)	74.2	5.2	65–84
Education (years)	12.8	2.2	9–16

### Materials

We selected the following tests for remote assessment: Raven’s Coloured Progressive Matrices (RCPM) [15], story recall, 10/36 spatial recall, selective reminding test, Symbol Digit Modalities Test (SDMT), Paced Auditory Serial Addition Test (PASAT), Frontal Assessment Battery (FAB), Trail Making Test-A (TMT-A), Trail Making Test-B (TMT-B), visual cancellation task, digit span, and tapping span.

We obtained research use permission for the RCPM from Pearson Asia Inc., under the condition that we would not digitise the stimulus booklet. Story recall was extracted from the Japanese version of the Rivermead Behavioural Memory Test (RBMT) [16] and the research use permission was obtained from the author and publisher. The 10/36 spatial recall, selective reminding test, SDMT, and PASAT (3-s version) were extracted from the Japanese version of the Brief Repeatable Battery of Neuropsychological Tests (BRB-N) [17], and the research use permission was obtained from the author. We also obtained research use permission for the FAB [18] from the author of the Japanese version.

Regarding the TMT-A, TMT-B, visual cancellation task, digit span, and tapping span, we created them ourselves as original tasks because we could not obtain permission from the authors and publishers of these standardised Japanese tests. We created new tasks of TMT for VTC assessment: TMT-A for connecting numbers (1 to 20) with lines, and TMT-B for connecting numbers and Japanese kana letters (あ to こ) alternately with lines. Given the prevalence of age-related visual and/or motor capability problems, the size of the numbers was increased by approximately 1.5 times (from a diameter of 14 mm to 20 mm) compared with the standardised Trail Making Test-Japanese edition (TMT-J) [19] to enhance visibility and facilitate line connections on the touchscreen monitor. Furthermore, the number of targets was reduced from 25 to 20. We adjusted the size of the numbers and letters so that the size of the stimuli on the monitor matched the stimuli on an A4 size paper (placed vertically), and matched the trail lengths of A and B. For the visual cancellation task, we placed the Arabic numerals in 10 rows and 20 columns on the A4 size paper randomly; each row had 5–7 targets (number 5 was assigned as the target), for a total of 60 targets. In the standardised visual cancellation task in the Clinical Assessment for Attention (CAT) [20], Arabic numerals are assigned in 6 rows and 52 columns on an A3 size paper randomly with 19 targets (number 3 was assigned as the target) in each row. In the self-created task, the size of the numbers was approximately twice as large (from 15 mm^2^ to 28 mm^2^) compared to the CAT. Additionally, the number of targets was reduced from 114 to 60. Changes in the TMT-A, TMT-B, and visual cancellation task were expected to reduce errors and shorten the required time because of the larger text and fewer targets. However, since we did not modify the procedure, the intrinsically necessary cognitive functions, such as spatial exploration and attention, remained unchanged. Therefore, we believe that we have been able to maintain the original purpose of the tests. For the digit span and tapping span, we created a random set of numbers with no duplicate numbers and no more than three consecutive numbers in a sequence, following a previous study [21]. We adopted the better performance in either of the two trials for each number in the digit span and tapping span tasks. The only difference between the standardised CAT's digit span and tapping span tasks [20] and the self-created digit span and tapping span tasks was the content of the number sequences. Therefore, there were no significant differences in the required cognitive function. To avoid adding alternate-form differences, we repeated the same exact tests in all tasks.

### Reliability of the self-created tests

The reliability of the self-created test was examined in a different group of 39 healthy older participants (16 men and 23 women) who were also recruited from senior citizen clubs and an employment service centre in Sapporo, Japan. Because five of the 39 participants had also entered in the VTC validation study, these five received these tests one year later to rule out learning effects. Their average age was 76.7 years (SD: 7.6; range: 64–90 years), with a mean RCPM score of 27.8 (SD: 3.8; range: 20–35). Their average number of years of education was 13.1 (SD: 1.8; range: 8–16 years) (Table 2). We examined the correlations between the self-created TMT and standardised TMT-J [19], and the self-created visual cancellation task and standardised visual cancellation task in the CAT [20].

**Table 2:** Demographics of the examination of the reliability of the self-created test (*n* = 39)

Variable	Mean	SD	Range
Age (years)	76.7	7.6	64–90
Education (years)	13.1	1.8	8–16

**Fig. 1:**
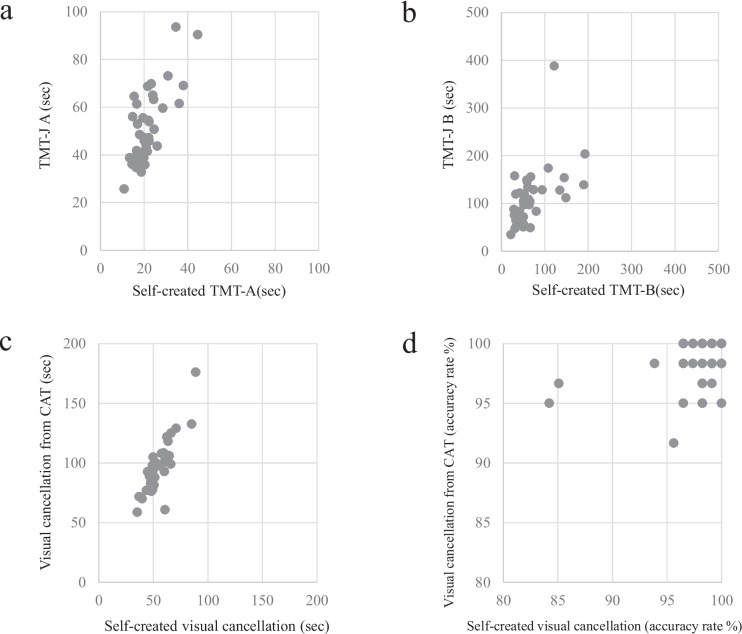
Scatter plot of self-created tests and standardised tests. **a** The scatter plot between the self-created TMT-A and standardized TMT-J A (*n* = 39). **b** The scatter plot between the self-created TMT-B and standardized TMT-J B (*n* = 39). **c** The scatter plot between the self-created visual cancellation task and standardized visual cancellation task (sec) (*n* = 39). **d** The scatter plot between the self-created visual cancellation task and standardized visual cancellation task (accuracy rate %) (*n* = 39)

The same examiner administered both tests on the same day, and the order of administration was counterbalanced among the participants. Pearson’s correlation coefficient was calculated. The result of TMT-A was r = 0.74 (*p* < 0.0001), TMT-B was r = 0.55 (*p* < 0.0003), time of the visual cancellation task was *r* = 0.85 (*p* < 0.0001), and accuracy rate of visual cancellation task was *r* = 0.36 (*p* < 0.025), each with a significant correlation. The details of the results are presented in Fig. 1. These findings indicated that the cognitive functions examined using standardised and self-created tasks demonstrated substantial consistency.

### Procedures

The experimental design was an open-label, counterbalanced, crossover, randomised controlled trial. We randomised participants into two groups by order of assessment administration (1:1 ratio): VTC-first and FTF-first. We used stratified block randomisation with age (< 65 years or ≧65 years) and gender as factors, and the block size was randomly varied between 2 and 4. However, there were no entries under the age of 65 years. An assistant not involved in the evaluation generated the random allocation sequence using web software [22] assignment, and informed the examiner after recruitment.

A nationally certified speech-language pathologist with at least 10 years of clinical experience conducted both, the VTC and FTF assessments, on the same day, with an interval of approximately 60 min. The FTF assessment took approximately 50 min, and the VTC assessment took approximately 60 min. The examiner conducted the VTC assessment remotely by controlling the participant’s PC from a separate room in the same building (central campus at Hokkaido University). No assistant was present during the assessment. We used Webex Meetings (Cisco Systems, Inc.) as a VTC system. We confirmed that the Internet speed was maintained at more than 70 Megabits per second (bps) for both downloads and uploads on all connected devices. We adapted the default value of Webex Meetings, 360 × 180, for image resolution because the goal of this study was a simple and low-cost implementation.

### Experimental equipment and setup

#### Tasks using only voice and video communication

We used voice and video calls for story recall, selective reminding test, PASAT, digit span, similarities (FAB), and word fluency (FAB). The examiner used a laptop PC (LIFEBOOK WU2/D2, 13.3-inch display manufactured by FUJITSU, Japan) with a unidirectional microphone (AT9933USB, manufactured by audio-technica) connected to a webcam (UCAM-C980FBBK, manufactured by ELECOM). The examiner also used Microsoft Groove music and the audio sharing function in Webex Meetings for the presentation of PASAT. Participants used a desktop PC (Gemini X45 manufactured by Beelink) with a 25-inch colour IPS monitor (manufactured by Philips), unidirectional microphone (same as the examiner), webcam (same as the examiner), and headphone (ATH-250 M manufactured by audio-technica). The distance between the participant and monitor was set at approximately 50 cm. Before the experiment began, the examiner checked the volume and adjusted it to a level that allowed each participant to hear the conversation comfortably. The participants did not operate any equipment other than wearing the headphones.

#### Motor-dependent tasks that require writing or drawing operations

For the 10/36 spatial recall, SDMT, TMT-A, TMT-B, and visual cancellation tasks, which require writing or drawing operations, we used Microsoft PowerPoint and the screen sharing function of Webex Meetings. PowerPoint ran on the examiner’s laptop PC and was projected onto the participant’s PC (ENVY × 360 13-ay1000 manufactured by HP) connected to a 23.8-inch touch monitor (P2418HT manufactured by Dell) using the screen sharing function of Webex Meetings. This touch monitor is equivalent to commonly available touch-panel displays and is not a special device. To practice the use of the touch monitor and stylus pen before the test started, we asked participants to write numbers and draw circles and lines. Participants wore gloves when malfunctions occurred because their palms touched the monitor. The participants did not operate any equipment other than holding the stylus pen and drawing. Additionally, none of the participants reported difficulty in operating the stylus pen.

In the VTC task, the testing seat was presented on the monitor of the same size as the FTF testing paper, and the examiner checked the handwriting locus in real-time on the examiner's monitor. In the 10/36 spatial recall, since participants could not use the checkers for answering, we asked participants to draw circles on the monitor-projected checkerboard. In 10/36 spatial recall using VTC, there is no limit to the number of checkers that can be placed; therefore, filling the checkerboards would result in a perfect score. Therefore, we defined this score as the number of correct answers minus the number of incorrect answers.

#### Motor-dependent tasks that require confirmation of hand movements by the examiner

**Fig. 2:**
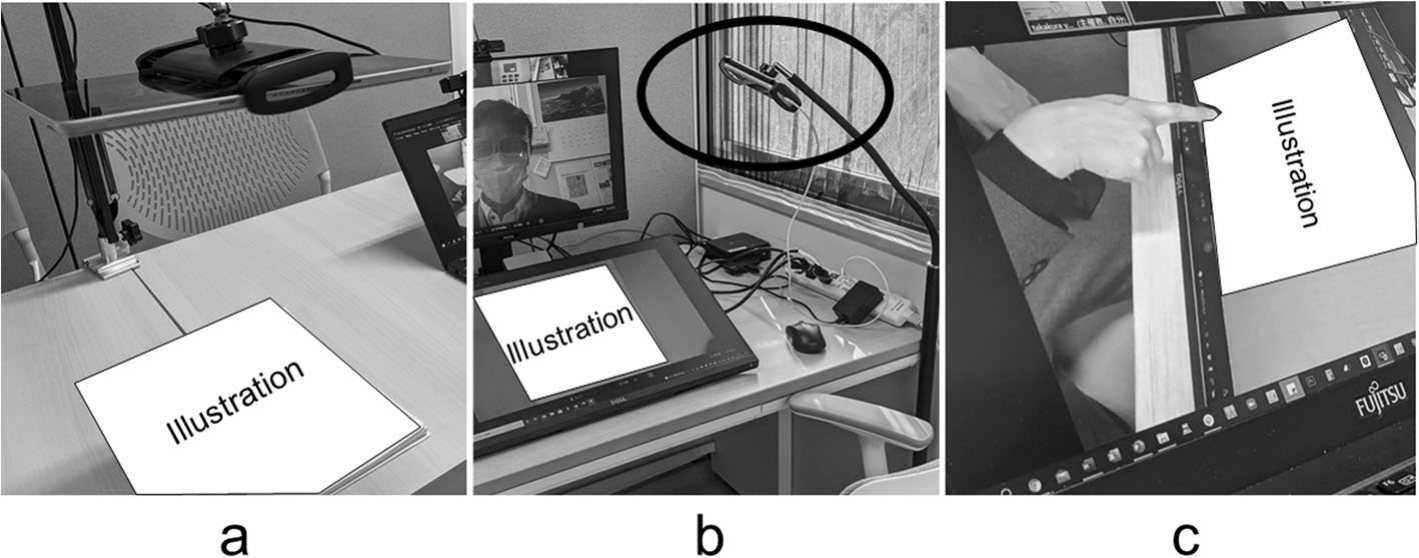
Situations of motor-dependent tasks that require confirmation of hand movements by the examiner. **a** The examiner projects the RCPM illustration using the tablet at the examiner’s booth. **b** The illustration is projected on the monitor at the participant’s booth. The circle indicates the webcam used to detect finger movements. **c** Checking the participant's pointing on the monitor at the examiner’s booth

For the RCPM, tapping span, motor series ‘Luria’ test (FAB), conflicting instructions (FAB), and go–no go (FAB), we used the webcam (Fig. 2a) built into the tablet (Surface Go manufactured by Microsoft) to show the examiner’s hand movement and illustrations on the participants’ monitor. The examiner checked the participant’s hand movement and pointing location through the webcam (Fig. 2b) built into the smartphone (iPhone 8 manufactured by Apple) in real time on the examiner’s monitor (Fig. 2c). Regarding the RCPM, unlike the original, we adopted the time required for the answer as the evaluation point, as well as the total score. The participants did not operate any equipment.

### Statistical analysis

A previous study recommended that the pilot study sample size should be 10% of the sample size of the project study [23]. The largest study of web-based neuropsychological assessment enrolled 202 participants [10]. Therefore, we estimated that 26 participants would be sufficient. Intraclass correlations (ICCs) and paired-samples t-tests were performed to compare the scores between the VTC and FTF conditions. We regarded ICC values as follows, based on Koo et al. [24]: < 0.50, poor; between 0.50 and 0.75, fair; between 0.75 and 0.90, good; above 0.90, excellent. Kappa coefficients and Wilcoxon signed-rank test were used for digit span and tapping span because the range of scores was narrow. We regarded κ as follows based on Ladies et al. [25]: between 0.00 and 0.20, slight; between 0.21 and 0.40, moderate; between 0.41 and 0.60, substantial; between 0.61 and 0.80, almost perfect; above 0.81, excellent. Analyses were performed using IBM SPSS Statistics 26, and the Holm method was used for multiple comparison correction (α level was 0.05).

### Incidents during VTC assessment

During the VTC assessment, there was one incident each of non-response of the touch monitor and failure to turn on the PowerPoint screen. Since assistants were not present, the examiner moved to the participant's room and performed troubleshooting. In each case, the problem was resolved within approximately two–three minutes, and the assessment was continued. In addition to these incidents, the experimenter did not encounter any technical issues during the assessments.

### An Institutional Review Board approval statement and statement of patient consent

The Ethics Committee of the Faculty of Health Sciences, Hokkaido University approved this study (approval number:20–21-1). All participants provided written informed consent. Furthermore, the Ethics Committee determined that this study was not recognised as an intervention study and that trial registration was not required.

## Results

All ICCs were significant and ranged from 0.47 (RCPM time) to 0.92 (RCPM score and PASAT), with a mean ICC of 0.75 (Table 3). In the reasoning domain, the RCPM scores demonstrated excellent concordance between the FTF and VTC conditions. However, the ICC was lower for the RCPM time, the lower bounds of the 95% CI were above zero and the confidence intervals were wide. In the memory domain, all scores showed significant concordance equivalent to ‘fair’ (ICC range: 0.65–0.73), and no noticeable differences were observed between verbal and spatial memory. In the frontal lobe function domain, despite the inclusion of motor-dependent tasks that required the confirmation of hand movements, the FAB scores demonstrated ‘good’ concordance (ICC = 0.79). In the attention domain, the PASAT, which is a synchronisation task, and the TMT, which is a synchronous and motor-dependent task, showed high concordance (PASAT’s ICC = 0.92, TMT-A’s ICC = 0.83, TMT-B’s ICC = 0.89). Additionally, the accuracy rates on the visual cancellation task showed ‘excellent’ concordance. However, the ICC was ‘fair’ for the visual cancellation time; the lower bounds of the 95% CI were above zero and the confidence intervals were wide. Although the SDMT also showed ‘fair’ concordance, the lower bounds of the 95% CI were close to zero and the confidence intervals were wide. Digit span using Cohen's kappa (κ) coefficient was significant, but the tapping span was not (Table 4). The paired samples t-test showed statistically significant differences in SDMT, RCPM time, and cancellation time (Table 5). These three items were characterised by a wide 95% CI. The Wilcoxon signed-rank test showed no statistically significant differences in digit span and tapping span (Table 6).

**Table 3:** Intraclass correlations between FTF and VTC conditions

Cognitive domain	Test	ICC	95%CI	*p*-value	Interpretation
Reasoning	RCPM score	0.923	0.811–0.967	< .001*	excellent
	RCPM time (sec)	0.472	-0.189–0.772	.006*	poor
Memory	Story—Immediate Recall (RBMT)	0.723	0.384–0.876	.001*	fair
	Story—delayed Recall (RBMT)	0.725	0.381–0.878	.001*	fair
	10/36 spatial recall immediate recall (BRB-N)	0.654	0.238–0.845	.005*	fair
	10/36 spatial recall delayed recall (BRB-N)	0.699	0.320–0.867	.002*	fair
	Selective reminding test (number of recalled words in 6 trials) (BRB-N)	0.646	0.203–0.843	.007*	fair
	Selective reminding test delayed recall (BRB-N)	0.679	0.274–0.858	.004*	fair
Frontal lobe functions	FAB (excluded prehension behaviour)	0.791	0.531–0.907	< .001*	good
Attention	SDMT (BRB-N)	0.685	0.050–0.879	< .001*	fair
	PASAT (BRB-N)	0.923	0.798–0.968	< .001*	excellent
	Trail making test—A time (sec)	0.829	0.624–0.923	< .001*	good
	Trail making test—B time (sec)	0.885	0.746–0.949	< .001*	good
	Cancellation task accuracy rate	0.919	0.819–0.964	< .001*	excellent
	Cancellation task time (sec)	0.722	-0.068–0.906	< .001*	fair

^*^significance in the intraclass correlation coefficient with Holm correction. Interpretations are as follows: < .50, poor; between .50 and .75, fair; between .75 and .90, good; above .90, excellent [21]. *Abbreviations*: *FTF* Face-to-Face, *VTC* video teleconference, *ICC* Intraclass Correlations, *CI* Confidence Interval, *RCPM* Raven’s Coloured Progressive Matrices, *RBMT* Rivermead Behavioural Memory Test, *BRB-N* Brief Repeatable Battery of Neuropsychological tests, *FAB* Frontal Assessment Battery, *SDMT* Symbol Digit Modalities Test, *PASAT* Paced Auditory Serial Addition Test

**Table 4:** Cohen’s Kappa coefficients between FTF and VTC conditions

Cognitive domain	Test	κ	*p*-value	Interpretation
Attention	Digit span forward	.438	< .001*	moderate
	Digit span backward	.390	.001*	fair
	Tapping span forward	.297	.053	-
	Tapping span backward	-.005	.671	-

^*^ = significance in Cohen’s kappa coefficient with Holm’s correction. Interpretations are as follows: < .00, poor; between .00 and .20, slight; between .21 and .40, fair; between .41 and .60, moderate; between .61 and .80, substantial; above 0.81, almost perfect [22]. *Abbreviations*: *FTF* Face-to-Face, *VTC* video teleconference

**Table 5:** Mean differences between FTF and VTC conditions

Cognitive domain	Test	FTF		VTC		r	*p*-value
		mean	SD	mean	SD		
Reasoning	RCPM score	29.5	4.6	30.6	4.5	0.16	.028
	RCPM time (sec)	234.7	95.8	339.3	100.5	0.05	< .001*
Memory	Story—Immediate Recall (RBMT)	10.8	3.3	11.2	2.6	0.2	.436
	Story—delayed Recall (RBMT)	9.3	3.7	9.5	2.7	0.03	.821
	10/36 spatial recall immediate recall (BRB-N)	6.4	11.4	8.5	9.3	0.09	.307
	10/36 spatial recall delayed recall (BRB-N)	2.1	4.3	2	4.3	0.02	.889
	Selective reminding test (number of recalled words in 6 trials) (BRB-N)	8.7	2	8.5	1.3	0.67	.651
	Selective reminding test delayed recall (BRB-N)	6.1	2.8	6.1	2.3	0.74	.939
Frontal lobe functions	FAB (excluded prehension behaviour)	12.1	1.7	12.2	1.6	0.08	.775
Attention	SDMT (BRB-N)	44.9	9.5	37.7	10.2	0.27	.001*
	PASAT (BRB-N)	38	15	34.5	12.7	0.18	.015
	Trail making test—A time (sec)	20.6	5.6	19.6	4.7	0.06	.180
	Trail making test—B time (sec)	52.6	26.6	55.9	31.7	0.47	.370
	Cancellation task accuracy rate (%)	98.8	2.5	98.7	3.5	0.42	.692
	Cancellation task time (sec)	48.9	7.4	55.7	9.7	0.73	< .001*

^*^ = significant difference in the paired t-test with Holm’s correction. *Abbreviations*: *FTF* Face-to-Face, *VTC* video teleconference, *SD* = standard deviation, *RCPM* Raven’s Colored Progressive. Matrices, *RBMT* Rivermead Behavioral Memory Test, *BRB-N* Brief Repeatable Battery of Neuropsychological tests, *FAB* Frontal Assessment Battery, *SDMT* Symbol Digit Modalities Test, *PASAT* Paced Auditory Serial Addition Test

**Table 6:** Median differences between FTF and VTC conditions

Cognitive domain	Test	FTF		VTC		R	*p*-value
		median	25–75 percentile	median	25–75 percentile		
Attention	Digit span forward	6	5–7	5	5–6.3	0.132	0.342
	Digit span backward	4	3–4	4	3–5	0.209	0.132
	Tapping span forward	6	6–6	6	6–6	0	1
	Tapping span backward	5	5–6	5	4–6	0.003	0.983

*Abbreviations FTF* Face-to-Face, *VTC* video teleconference

## Discussion

In this study, we detected significant concordance for all tests, except for the tapping span. Some tests, such as the RCPM, PASAT, and TMT-B, showed good or excellent agreement. The results on the RCPM showed that it is possible to remotely assess intellectual functions for which there is little evidence. Additionally, our results showed that the PASAT, which is a synchronisation task, and the TMT, which is a synchronous and motor-dependent task, can be evaluated remotely, even with common equipment and software. On the other hand, previous studies have indicated that remote assessment may have a negative effect on synchronous tasks, and that movement-dependent tasks are heterogeneous [11]. The differences observed in our study may be attributed to the potential influence of internet connection speed. Among the studies analysed by Brearly et al. [11], seven out of eleven studies utilised communication speeds below 364 kbps. Our investigation ensured a connection speed of 70 Mbps (1 Mbit is equivalent to 1000 kbits), which is speculated to have contributed to the high concordance observed in real-time synchronous and motor-dependent tasks. However, there were significant differences in the results for the required speed processing tasks, that is, RCPM (time), SDMT, and visual cancellation tasks (time), all of which showed a time delay or low number of achievements within the time limit in VTC conditions. We suspect that the delay in the SDMT and visual cancellation tasks was caused by a lack of familiarity with the fine writing action with the stylus pen. In the RCPM using VTC, because the RCPM was not digitised, the examiner had to present each illustration to the webcam (Fig. 2a), ensuring that the illustration appeared correctly on the participant’s monitor (Fig. 2b). We speculate that the time delay in the RCPM may have been caused by operational delays by the examiner. These problems can be solved by setting a time baseline for the VTC conditions.

Next, we considered factors that did not show agreement in the tapping span. Since no significant differences were found in the scores, it is unlikely that there was a difference in difficulty between the VTC and FTF conditions. One possibility is the instability of the test itself. Among the 16 items of the Japanese standard attention test in the CAT, the test–retest reliability of the tapping span (forward) was the lowest (ICC = 0.69) [21]. Therefore, we believe that this disagreement can be eliminated by increasing the number of test trials.

This study has important implications. The most clinically feasible application is the use of the neurocognitive assessment for patients hospitalised during pandemics or other situations that require infection prevention measures. This is because the present study demonstrated reliability under the condition of ‘different rooms within the same building’. Additionally, it can be implemented in hospitals using inexpensive devices. However, remote assessments connecting medical facilities to participants’ homes are impractical because of extensive device setup requirements. When targeting older people, it is advisable to avoid procedures that require them to operate devices. Nevertheless, an application similar to this study, in which examination booths are established with pre-installed equipment that is remotely operated by examiners, could potentially enable cognitive function monitoring by connecting specialised hospitals with facilities accommodating older adults or community centres in the region.

Finally, we acknowledge the limitations of this study and suggest avenues for future research. As pointed out in previous reports of similar designs [26], the limitations of this study include small sample sizes, short intervals between test–retest conditions, and the same examiner’s presence for both test conditions may have enhanced the observed correlations. Furthermore, our study included only healthy participants, and it is unclear whether similar agreements can be found in cases of Mild Cognitive Impairment (MCI) and dementia. Additionally, since there was only one examiner in this study, it is essential to confirm the inter-rater reliability by involving multiple examiners. Finally, in the setting of this study, an assistant may be required to manage troubleshooting in the case of remote assessments that connect different facilities. Therefore, future research should focus on the following aspects: (1) standardisation of assessments specifically designed for remote evaluations, (2) validation of these assessments in different populations, such as individuals with MCI or dementia, (3) establishment of inter-rater reliability among different examiners, and (4) evaluation of remote testing connecting different facilities. Additionally, our study did not systematically investigate the participants’ perspectives and experiences. Previous reports have generally reported high satisfaction with remote assessments using the MoCA-J [4]. However, their satisfaction levels may vary depending on the specific content of the neuropsychological tests. Therefore, investigating satisfaction levels for each type of assessment remains an important area for future research.

## Conclusions

Our results suggest that remote assessment with common Information and Communication Technology (ICT) devices and software can be used to assess a wide range of cognitive domains in healthy older adults. For processing speed tasks, we need to create our own standards for the VTC condition. For the tapping span that showed inconsistency, we speculated that it would be necessary to increase the number of trials. We believe that this pilot study will encourage further research to improve medical access, the imbalance between supply and demand due to the shortage of examiners, and the difficulty of implementing tele-neuropsychological assessments in clinical practice in Japan.

## Data Availability

The datasets used and/or analysed during the current study are available from the corresponding author upon reasonable request.

## Abbreviations

VTC: video teleconference
HDS-R: Revised Hasegawa's dementia scale
MoCA-J: Japanese version of Montreal Cognitive Assessment
ADAS-cog: Alzheimer's Disease Assessment Scale-cognitive component
FTF: face-to-face
SD: standard deviation
RCPM: Raven’s Coloured Progressive Matrices
SDMT: Symbol Digit Modalities Test
PASAT: Paced Auditory Serial Addition Test
FAB: Frontal Assessment Battery
TMT-A: Trail Making Test-A
TMT-B: Trail Making Test-B
RBMT: Rivermead Behavioural Memory Test
BRB-N: Brief Repeatable Battery of Neuropsychological tests
TMT-J: Trail Making Test-Japanese edition
CAT: Clinical Assessment for Attention
ICCs: Intraclass correlations
MCI: Mild Cognitive Impairment
ICT: Information and Communication Technology
